# Supplementary material for: From sequence to enzyme mechanism using multi-label machine learning
Source: BMC Bioinformatics. 2014 May 19;15:150. doi: 10.1186/1471-2105-15-150 (PMC4229970; doi:10.1186/1471-2105-15-150)
Supplement: Additional file 2 — Java code of ml2db. Additional file ml2db_code.tar.gz contains the Java source code to run the multi-label machine learning experiments and save the results to database. The code’s Javadoc is included. [file 1471-2105-15-150-S2.zip › additional file 2/ml2db/utils/doc/index-files/index-8.html]

H-Index


JavaScript is disabled on your browser.


- Overview
- Package
- Class
- Use
- Tree
- Deprecated
- Index
- Help

- Prev Letter
- Next Letter

- Frames
- No Frames

- All Classes

A B C D E F G H I J K L M N O P Q R S T U V W X Y 


## H

hasChildren() - Method in class uk.ac.standrews.utils.main.webutils.simpledomparser.XmlNode
:   Whether a SimpleElement node has child nodes.

hasKey() - Method in class uk.ac.standrews.utils.main.database.Table
:   Gets the "has primary key" status for the table

histogram(TreeMap<Integer, Integer>) - Static method in class uk.ac.standrews.utils.main.stats.StatUtils
:   Returns a frequency histogram

histogramBar(Integer, Integer) - Static method in class uk.ac.standrews.utils.main.stats.StatUtils
:   Returns an histogram bar

HOST\_PROP - Static variable in class uk.ac.standrews.utils.main.database.DbConn


HOUR - Static variable in class uk.ac.standrews.utils.main.TimeUtils

A B C D E F G H I J K L M N O P Q R S T U V W X Y

- Overview
- Package
- Class
- Use
- Tree
- Deprecated
- Index
- Help

- Prev Letter
- Next Letter

- Frames
- No Frames

- All Classes
